# Neutrophil-to-lymphocyte and platelet-to-lymphocyte ratios and prognosis after aneurysmal subarachnoid hemorrhage: a cohort study

**DOI:** 10.1055/s-0043-1768662

**Published:** 2023-06-28

**Authors:** Adilson Jose Manuel de Oliveira, Nicollas Nunes Rabelo, João Paulo Mota Telles, Davi Jorge Fontoura Solla, Antonio Carlos Samaia da Silva Coelho, Guilherme Bitencourt Barbosa, Natália Camargo Barbato, Marcia Harumy Yoshikawa, Manoel Jacobsen Teixeira, Eberval Gadelha Figueiredo

**Affiliations:** 1 Universidade de São Paulo, Hospital das Clínicas, Faculdade de Medicina, Divisão de Neurocirurgia, São Paulo SP, Brazil.; 2 Clínica Girassol, Divisão de Neurocirurgia, Luanda, Angola.; 3 Universidade de São Paulo, Hospital das Clínicas, Faculdade de Medicina, Divisão de Neurologia, São Paulo SP, Brazil.; 4 Universidade de São Paulo, Hospital das Clínicas, Faculdade de Medicina, São Paulo SP, Brazil.; 5 Centro Universitário Saúde ABC, Faculdade de Medicina do ABC, Santo André SP, Brazil.

**Keywords:** Aneurysm, Neutrophils, Lymphocytes, Platelet Count, Subarachnoid Hemorrhage, Aneurisma, Neutrófilos, Linfócitos, Contagem de Plaquetas, Hemorragia Subaracnóidea

## Abstract

**Background** Subarachnoid hemorrhage (SAH) prognosis remains poor. Vasospasm mechanism might be associated with inflammation. Neutrophil-to-lymphocyte ratio (NLR) and platelet-to-lymphocyte ratio (PLR) have been studied as inflammation markers and prognostic predictors.

**Objective** We aimed to investigate NLR and PLR in admission as predictors of angiographic vasospasm and functional outcome at 6 months.

**Methods** This cohort study included consecutive aneurysmal SAH patients admitted to a tertiary center. Complete blood count was recorded at admission before treatment. White blood cell count, neutrophil count, lymphocyte count, platelet count, NLR, and PLR were collected as independent variables. Vasospasm occurrence-modified Rankin scale (mRS), Glasgow outcome scale (GOS), and Hunt-Hess score at admission and at 6 months were recorded as dependent variables. Multivariable logistic regression models were used to adjust for potential confounding and to assess the independent prognostic value of NLR and PLR at admission.

**Results** A total of 74.1% of the patients were female, with mean age of 55.6 ± 12.4 years. At admission, the median Hunt-Hess score was 2 (interquartile range [IQR] 1), and the median mFisher was 3 (IQR 1). Microsurgical clipping was the treatment for 66.2% of the patients. Angiographic vasospasm incidence was 16.5%. At 6 months, the median GOS was 4 (IQR 0.75), and the median mRS was 3 (IQR 1.5). Twenty-one patients (15.1%) died. Neutrophil-to-lymphocyte ratio and PLR levels did not differ between favorable and unfavorable (mRS > 2 or GOS < 4) functional outcomes. No variables were significantly associated with angiographic vasospasm.

**Conclusion** Admission NLR and PLR presented no value for prediction of functional outcome or angiographic vasospasm risk. Further research is needed in this field.

## Resumo

**Antecedentes** O prognóstico da hemorragia subaracnoidea (HSA) permanece ruim. Vasoespasmo pode estar associado à inflamação. Razões neutrófilo-linfócito (NLR) e plaqueta-linfócito (PLR) têm sido estudadas como marcadores de inflamação e prognóstico.

**Objetivo** Investigar NLR e PLR na admissão como preditores de vasoespasmo angiográfico e desfecho aos 6 meses.

**Métodos** Este estudo de coorte incluiu pacientes consecutivos com HSA aneurismática de um centro terciário. Contagem de leucócitos, neutrófilos, linfócitos e plaquetas, proporção de neutrófilos para linfócitos e de plaquetas para linfócitos foram coletados como variáveis independentes. Ocorrência de vasoespasmo, escala de Rankin modificada, escala de desfecho de Glasgow e o escore de Hunt-Hess na admissão e 6 meses após a mesma foram registradas como variáveis dependentes. Modelos de regressão logística multivariável foram usados para ajustar potenciais fatores de confusão e avaliar valor prognóstico independente de NLR e PLR.

**Resultados** Um total de 74,1% pacientes eram do sexo feminino, com idade média de 55,6 ± 12,4 anos. Na admissão, a pontuação média de Hunt-Hess foi de 2 (IQR 1) e a mediana de mFisher foi de 3 (IQR 1). Clipagem microcirúrgica foi o tratamento escolhido para 66,2% dos pacientes. A incidência de vasoespasmo angiográfico foi de 16,5%. Aos 6 meses, a escala de desfecho de Glasgow mediana era 4 (IQR 0,75) e a escala de Rankin modificada mediana era 3 (IQR 1,5). Vinte e um pacientes (15,1%) morreram. Os níveis de NLR e PLR não diferiram entre resultados funcionais favoráveis e desfavoráveis (mRS > 2 ou GOS < 4). Nenhuma variável foi significativamente associada ao vasoespasmo angiográfico.

**Conclusão** Razão neutrófilo-linfócito e a PLR não apresentaram valor preditivo de desfecho funcional ou risco de vasoespasmo angiográfico. Mais pesquisas são necessárias neste campo.

## INTRODUCTION

Ruptured intracranial aneurysms are the leading cause of non-traumatic subarachnoid hemorrhage (SAH), a medical emergency with high morbidity and mortality accounting for ∼ 5% of all cerebrovascular deaths. 1 2 Cerebral vasospasm following SAH is the most important cause of delayed cerebral ischemia, neurologic impairments, and deaths. The diagnosis of vasospasm is both clinical and angiographic. Although angiographic vasospasm may be observed in almost 70% of patients, only 20 to 30% present clinical manifestations. 3 4 Despite advances in diagnosis and treatment, SAH and vasospasm prognoses remain very poor, with mortality approaching 50%. 5 6

Proinflammatory agents, such as talc (crystallized hydrogen sulfate), latex, polystyrene and dextran beads, lipopolysaccharide (LPS), and tenascin C, when administered intracisternally, led to vasospasm. 7 This fact indicates that vasospasm can occur in the absence of blood (red blood cells and hemoglobin) and corroborates the pathophysiologic theory including inflammation. 8

Previous studies demonstrate low evidence of inflammatory cascade in vasospasm and antiinflammatory and antiplatelet treatments of SAH. In the last decade, novel systemic inflammation markers have been studied. Neutrophil-to-lymphocyte ratio (NLR) and platelet-to-lymphocyte ratio (PLR) are widely available at a low cost and have shown a potential prognostic role in several conditions, such as sepsis, cancer, and acute coronary syndromes. 9 10 11

In neurosurgery, NLR and PLR may be useful as prognostic factors for intracerebral hemorrhage, gliomas, and brain metastasis prognostication. 12 13 14 The proinflammatory effects of systemic inflammatory response (SIR) have been linked with those conditions. Increasing inflammatory response results in upregulation of cytokines and inflammatory mediators, inhibition of apoptosis, angiogenesis, and DNA damage.

The aim of this study is to investigate the admission NLR and PLR as prognostic markers for vasospasm, functional outcome, and mortality in patients with SAH due to ruptured cerebral aneurysms.

## METHODS

### Patient selection

This cohort study with prospective evaluation of outcome and retrospective data collection of NLR and PLR was conducted between January 2018 and December 2019.

The inclusion criteria were the following: aneurysmal SAH confirmed by computed tomography (CT) or cerebrospinal fluid puncture in case of a negative CT; presence of cerebral aneurysm confirmed by angiotomography or digital subtraction angiography (DSA); initial blood sampling for laboratory test at admission and patient age >18 years.

We excluded patients with acute or chronic infection, previous autoimmune disease, previous or recent cerebrovascular disease, previous use of anticoagulant/antiplatelet medication, malignancy, uremia, liver cirrhosis, chronic heart disease, and chronic lung disease. Patients who suffered aneurysm rebleeding before treatment or declined surgical or endovascular intervention were also ineligible.

This research project was approved by the Ethics and Research Committee of Hospital das Clínicas, Faculdade de Medicina da Universidade de São Paulo. Online registration CAPPesq: 15226 approved 06/20/2016. Approved on the Brazil platform CAAE number: 61719416.6.0000.0068.

### Clinical variables and outcomes

Hematologic tests of complete blood count, including white blood cell (WBC) count, neutrophil count (NC), lymphocyte count (LC), and platelet count (PC), were obtained from the hospital automatic test system (Sysmex KX-21 N Automated Hematology Analyzer, Sysmex America, Inc., Lincolnshire, IL, USA) and were collected at admission. Neutrophil-to-lymphocyte ratio (NLR) and PLR were calculated.

The patients' clinical severity was assessed through the Hunt Hess score. Radiological severity was reflected by the modified Fisher scale from the admission head CT, the presence of midline shift, and hydrocephalus. Digital subtraction angiography provided information regarding angiographic vasospasm in the CT scan. Therefore, vasospasm was only considered when confirmed by DSA.

The treatment modality was decided according to our multidisciplinary department protocol as well as patients' preference. After aneurysm treatment, patients received standard treatment according to international SAH management guidelines. 15 The primary outcome was the functional one at 6 months after SAH, which was evaluated at the outpatient service through the modified Rankin scale (mRS) and the Glasgow outcome scale (GOS). Angiographic vasospasm and mortality were secondary outcomes. There was no missing data.

### Statistical analysis

Continuous data are presented as mean (±standard deviation) or median (interquartile range), according to normality tests. Categorical data are presented as counts (valid percentages). Functional outcomes were analyzed as favorable (mRS ≤ 2 or GOS ≥ 4) or unfavorable (mRS > 2 or GOS < 4). The NLR and PLR between groups were compared using the Mann-Whitney U test. Logistic regressions were modeled to assess the association of NLR, PLR, and other potential covariates with the outcomes. Covariates were included in the final multivariable models if presenting a *p* -value < 0.1 in the univariate analyses. Moreover, due to biological plausibility, age, Glasgow coma scale and Hunt-Hess were included for the models evaluating functional outcomes, and age and modified Fisher scale were included for the models evaluating the risk of vasospasm. Correlations between NLR and PLR with mortality at 6 months were analyzed using logistic regressions. All analyses were conducted using the R software (R Foundation for Statistical Computing, Vienna, Austria).

Angiographic vasospasm was defined as moderate (30–50%) or severe (> 50%) luminal narrowing on DSA not attributable, catheter-induced spasm, or vessel hypoplasia, as determined by the neuroradiology team in admission. No minimum number of altered vessels are needed for the definition of SAH-associated vasospasm. All patients were evaluated for vasospasm occurrence at admission with CT scan.

## RESULTS

### Patient characteristics

A total of 224 patients were assessed for eligibility, and 85 were excluded according to the prespecified exclusion criteria. The study included 139 patients, 103 of whom (74.1%) were female. The patients' mean age was 55.6 (±12.4) years. At admission, the median Hunt-Hess score was 2 (IQR 1), median GCS was 14 (IQR 6), and median mFisher was 3 (IQR 1). Microsurgical clipping was the definite treatment for 66.2% of patients, while 33.8% were treated endovascularly. Initial laboratory workup, including NLR and PLR values, are detailed in Table 1 .

**Table 1:** Patient characteristics

Variables		Total ( *n* = 139)
Age		55.6 (±12.4)
Female		103 (74.1%)
Platelets (thousand)		246 (±78.3)
Neutrophil		10.2 (±4.4)
Lymphocyte		1.76 (±0.99)
NLR		5.47 (7.98)
PLR		200.8 (117.6)
Treatment	Microsurgery	92 (66.2%)
	Endovascular	47 (33.8%)
Hunt-Hess scale		2 (1)
Glasgow coma scale		14 (6)
mFisher scale		3 (1)
Vasospasm (radiological)		23 (16.5%)
GOS 6 months		4 (0.75)
Rankin 6 months		3 (1.5)
Mortality		21 (15.1%)

Abbreviation: GOS, Glasgow outcome scale; NLR, neutrophil-to-lymphocyte ratio; PLR, platelet-to-lymphocyte ratio.

Notes: Data are presented as mean (±standard deviation), median (interquartile range), or count (valid percentage), as appropriate.

### Outcomes

**Figure 1:**
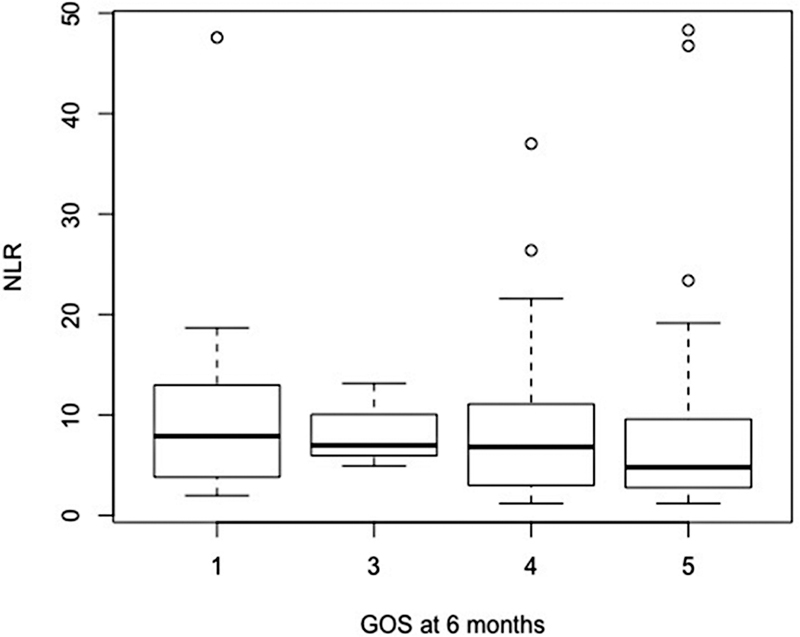
Abbreviation: GOS, Glasgow outcome scale; NLR, neutrophil-to-lymphocyte ratio. Functional outcome using GOS and NLR at admission.

**Figure 2:**
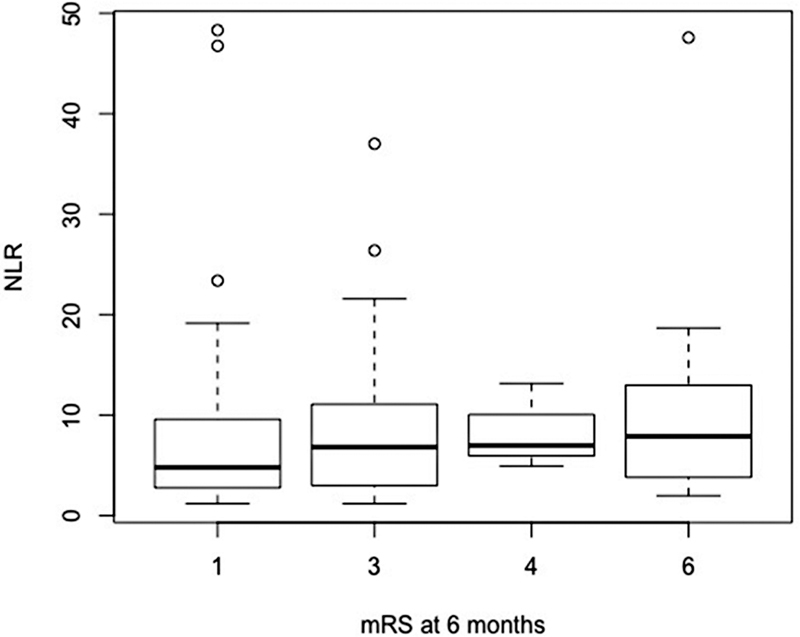
Abbreviation: NLR, neutrophil-to-lymphocyte ratio. Functional outcome using mRS and NLR at admission.

**Figure 3:**
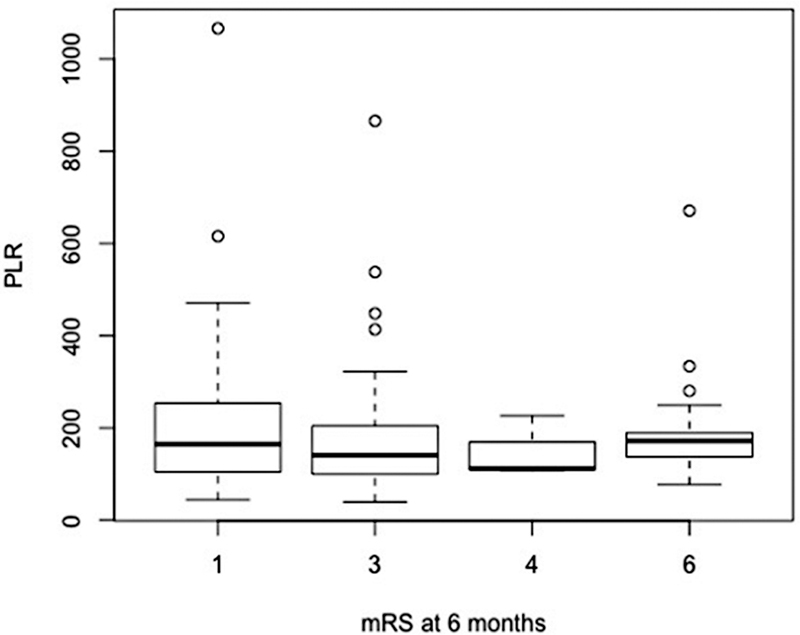
Abbreviations: PLR, platelet-to-lymphocyte ratio. Functional outcome using mRS and PLR at admission.

**Figure 4:**
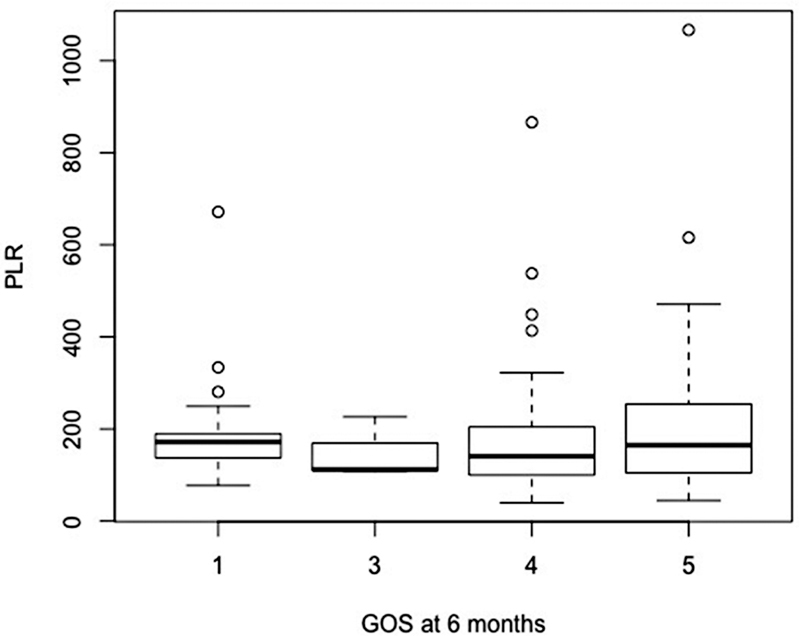
Abbreviation: GOS, Glasgow outcome scale; PLR, platelet-to-lymphocyte ratio. Functional outcome using GOS and PLR at admission.

**Figure 5:**
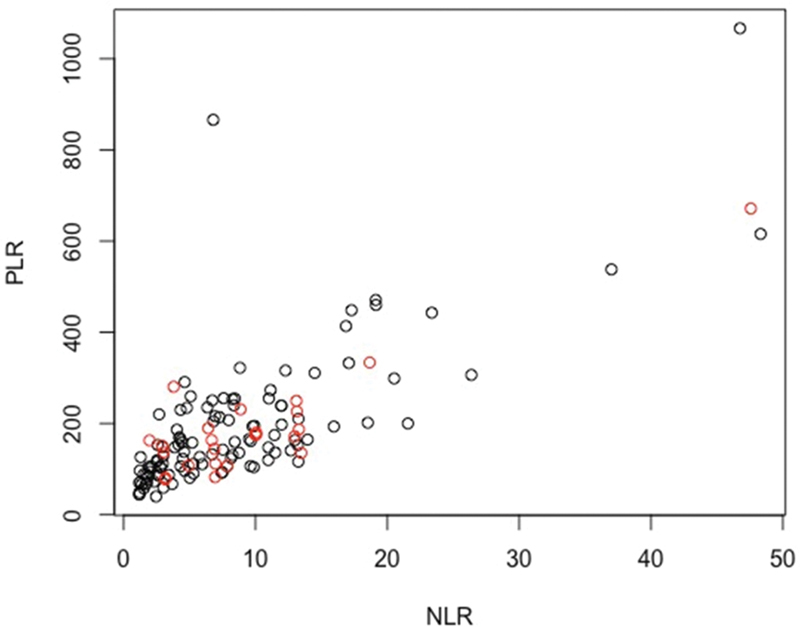
Abbreviation: GOS, Glasgow outcome scale. Notes: Black = favorable (GOS ≥ 4); red = unfavorable (GOS < 4). Dispersion representation of outcomes using GOS.

The incidence of vasospasm at admission, evaluated by CT scan, was 16.5%. At 6 months, the median GOS was 4 (IQR 0.75), and median mRS was 3 (IQR 1.5). A total of 21 patients (15.1%) died ( Figures 1 , 2 , 3 , 4 , and 5 ).

### Univariable analysis

Univariable analyses are shown in Table 2 . Endovascular treatment was associated with unfavorable functional outcomes as measured by the mRS (unfavorable mRS > 2). Admission GCS, mFisher, and platelet count were significantly associated with unfavorable functional outcomes as measured by the GOS (unfavorable GOS < 4). No variables were associated with angiographic vasospasm.

**Table 2:** Factors associated with 6-month functional outcomes – univariable

Variables	Rankin > 2			GOS < 4		
	OR	95% CI	P value	OR	95% CI	*P* -value
Age	1.02	0.99–1.05	0.25	0.62	0.24–1.67	0.33
Sex (female)	1.20	0.51–2.9	0.61	1.03	0.99–1.07	0.17
GCS	0.94	0.85–1.04	0.25	0.92	0.84–1.01	0.08*
HH	1.30	0.93–1.88	0.15	1.25	0.87–1.78	0.21
mFisher	1.01	0.72–1.39	0.93	1.56	1.00–2.74	0.08*
Midline shift	0.93	0.79–1.10	0.35	1.13	0.95–1.33	0.13
Vasospasm	1.61	0.55–8.90	0.42	0.67	0.15–2.19	0.55
Endovascular (versus microsurgery)	3.01	1.21–8.63	0.03*	1.83	0.74–4.50	0.18
Platelets (*1000)	0.99	0.99–1.00	0.61	0.99	0.99–1.00	0.07*
Neutrophil	1.05	0.96–1.15	0.31	1.06	0.96–1.17	0.23
Lymphocyte	0.88	0.6–1.3	0.49	0.73	0.41–1.18	0.23
NLR	0.99	0.94–1.03	0.55	1.02	0.97–1.07	0.44
PLR	0.99	0.99–1.0	0.1	1.0	0.99–1.0	0.98

Abbreviations: CI, confidence interval; NLR, neutrophil-to-lymphocyte ratio; OR, odds ratio; PLR, platelet-to-lymphocyte ratio.

Note: Logistic regressions.

### Multivariable analysis

As stated in the methods section, the covariates significantly associated with the outcome of interest in the univariable analyses were included in the multivariable models ( Table 3 ), except for platelets in the models that included PLR due to collinearity. For the mRS defined unfavorable functional outcome (mRS > 2), only endovascular treatment (compared with microsurgery) was associated with worse outcomes. For the GOS defined outcome (unfavorable GOS < 4), only admission GCS was independently associated ( Table 4 ). No variables were significantly associated with angiographic vasospasm ( Table 5 ).

**Table 3:** Multivariable analyses

Variables		OR	95% CI	*P* -value
mRS > 2	Intercept	1.0	0.16–6.30	0.99
	Age	1.02	0.98–1.05	0.33
	Endovascular	2.81	1.11–8.16	0.04*
	NLR	0.99	0.94–1.05	0.51
	Intercept	0.94	0.14–6.12	0.95
	Age	1.02	0.98–1.05	0.34
	Endovascular	2.80	1.11–8.10	0.04
	PLR	0.99	0.99–1.00	0.99
GOS < 4	Intercept	0.46	0.01–21.1	0.69
	Age	1.01	0.97–1.06	0.54
	GCS	0.89	0.80–0.99	0.03*
	Platelets	1.00	0.99–1.00	0.34
	mFisher	1.26	0.78–2.25	0.38
	NLR	0.99	0.92–1.04	0.65
	Intercept	0.21	0.01–5.79	0.37
	Age	1.02	0.98–1.06	0.43
	GCS	0.88	0.79–0.97	0.01
	mFisher	1.31	0.82–2.31	0.29
	PLR	1.0	0.99–1.0	0.30

Abbreviations: CI, confidence interval; GOS, Glasgow outcome scale; NLR, neutrophil-to-lymphocyte ratio; OR, odds ratio; PLR, platelet-to-lymphocyte ratio.

**Table 4:** Analysis of variation of NLR and PLR

Variables		GOS < 4		mRS > 2	
		OR	95% CI	OR	95% CI
Univariate	NLR 2 > NLR 1	0.40	0.11–1.15	0.51	0.23–1.17
	Delta NLR	0.97	0.93–1.02	0.99	0.94–1.03
	PLR 2 > PLR 1	0.85	0.34–2.05	0.62	0.28–1.36
	Delta PLR	1.0	0.99–1.00	1.0	0.99–1.0
Multivariable	NLR2 > NLR1	0.41	0.11–1.17	0.50	0.22–1.14
	Age	1.03	0.99–1.07	1.02	0.99–1.06
	PLR 2 > PLR 1	0.88	0.35–2.15	0.63	0.28–1.37
	Age	1.03	0.99–1.07	1.02	0.99–1.06
	Delta NLR	0.98	0.93–1.02	0.99	0.93–1.03
	Age	1.02	0.99–1.07	1.02	0.99–1.06
	Delta IPL	1.0	0.99–1.0	0.99	0.99–1.0
	Age	1.02	0.99–1.07	1.02	0.99–1.06

Abbreviations: CI, confidence interval; NLR, neutrophil-to-lymphocyte ratio; OR, odds ratio; PLR, platelet-to-lymphocyte ratio.

Notes: Logistic regressions evaluating the influence of the variations in NLR and PLR levels, both absolute (delta) or increase (ratio 2 > 1) versus no increase in ratios, on the probability of unfavorable clinical outcomes.

**Table 5:** Factors associated with vasospasm

Variables		OR	95% CI	*P* -value
Univariate	Sex	1.7	0.6–4.3	0.29
	Age	0.99	0.96–1.03	0.85
	Hunt Hess	0.94	0.63–1.36	0.76
	GCS	1.08	0.96–1.23	0.24
	Endovascular (versus microsurgery)	0.83	0.30–2.12	0.71
	Neutrophil	1.04	0.94–1.15	0.43
	Lymphocyte	0.92	0.55–1.43	0.72
	NLR	1.0	0.93–1.05	0.90
	PLR	1.0	0.99–1.00	0.47
	Mod Fisher	0.87	0.62–1.27	0.45
Multivariable	Intercept	0.24	0.03–1.83	0.18
	Age	1.0	0.96–1.03	0.86
	NLR	0.99	0.93–1.05	0.91
	Intercept	0.19	0.02–1.51	0.13
	Age	1.0	0.96–1.03	0.87
	PLR	1.0	0.99–1.0	0.47

Abbreviations: CI, confidence interval; GCS, Glasgow coma scale; neutrophil-to-lymphocyte ratio; OR, odds ratio, PLR, platelet-to-lymphocyte ratio.

Note: Logistic regressions.

### Admission NLR and PLR

Neutrophil-to-lymphocyte ratio levels did not differ between patients with favorable or unfavorable functional outcomes (GOS dichotomized: median 6.7, IQR 8.1 vs 7.4, IQR 8.4, respectively, *p* = 0.18; mRS dichotomized: median 4.8, IQR 6.8 vs median 6.9, IQR 8.1, respectively, *p* = 0.52.

Platelet-to-lymphocyte ratio levels were also similar between patients with favorable (median 147.9, IQR 124.1) or unfavorable (median 167.6, IQR 69.3) outcomes according to the GOS ( *p* = 0.43). The same held true using the mRS ( *p* = 0.29), comparing favorable (median 163.7, IQR 149.4) and unfavorable (148.1, IQR 98.9) outcomes.

Dichotomic analyses using multiple NLR cutoffs from 4 to 12 were attempted, but also failed to demonstrate a significant association.

Neutrophil-to-lymphocyte ratio was not associated with mortality at 6 months follow-up (OR 1.02, 95% CI 0.97–1.07, *p* = 0.34), nor was PLR (OR 0.99, 95% CI 0.99–1.01, *p* = 0.64).

## DISCUSSION

Neutrophil-to-lymphocyte ratio has been recently used as a biomarker of inflammation, especially in neurosurgery, for which there is no published research before 2013. 16 The current understanding points to a multifactorial process involving the NLR, including direct and reactive inflammation, immune dysregulation, and production of reactive oxygen species. 17

Prognosis in SAH is a very important issue, and trying to find a low cost and no morbidity associated biomarker as NLR is very attractive. Previous studies tried to use NLR as a biomarker of mortality, rebleeding, and vasospasm in SAH. 12 17 18 19 20

The identification of patients at high risk of progressing to death is of great importance in the treatment of critical cases. Thus, the identification of accurate, effective, inexpensive, and practical biomarkers may generate great progress in this field. 21 Neutrophil-to-lymphocyte ratio, monocyte-to-lymphocyte ratio (MLR), PLR, and mean platelet volume-to-platelet count (MPV/PC) ratio are parameters readily available. 22 Neutrophil-to-lymphocyte ratio and PLR have already been identified as indicators of systemic inflammation and worse overall survival (OS) in oncology. 23

Various studies have been performed in different clinical scenarios in which such markers can be used as predictors of the patient's outcome: MPV, NLR, and PLR support in the diagnosis of inflammation secondary to poisonous snake bite. 24 Mean platelet volume and NLR were reported to be independent predictors for mortality in bacteremia. 25 Neutrophil-to-lymphocyte ratio is linked to secondary damage caused by neutrophils and their products in brain tissue and indicates prognosis for patients with traumatic brain injury (lower GCS scores and higher mortality). 26 High NLR values are related to an increase in the number and severity of organ failure in trauma patients 27 ; preeclampsia is more common in patients with increased NLR and PLR 28 ; PLR and NLR reveal worse prognosis in the short and long term for patients with acute pulmonary embolism (PE). 29 Platelet-to-lymphocyte ratio tends to be reduced in hyperglycemic patients 30 ; there is a direct correlation between NLR and PLR with rheumatoid arthritis (RA). 31 In addition, there is linear relationship between NLR and a higher number of postoperative complications and, consequently, longer hospitalizations. 32 Neutrophil-to-lymphocyte ratio and PLR are useful to assess the activity of systemic lupus erythematosus disease (SLE) 33 34 ; NLR is recognized as an inflammation marker and as a prognostic factor in cases of metastatic castration-resistant prostate cancer (mCRPC). 35

Few studies have evaluated the role of NLR and PLR as outcome predictors in SAH, all of them retrospective in design. Tao et al. 12 and Wu et al. 20 studied the relationship between NLR and delayed cerebral ischemia with an area under the curve (AUC) of 0.64816 and 0.72217, and cutoff values of 14.3 and 11.47, respectively. Both studies had an acceptable AUC (0.6–0.8), mean AUC of 0.685, and mean cutoff value of 12.85. The mean sensitivity and specificity were 66.3% and 75.8%, respectively. Giede-Jeppe et al. 8 18 and Tao et al. 12 studied the relationship between NLR and functional outcome at 3 months; the studies showed an AUC of 0.614 and 0.700, respectively. Giede-Jeppe et al., 18 using a cutoff of 7.05, achieved a sensitivity of 61.4% and specificity of 59.6%. Tao et al. 12 found a sensitivity of 87.3% and specificity of 48.4% through a cutoff of 14.0.

Tao et al. ^12^ described mRS after 90 days of follow-up. Conversely, our paper has studied outcomes after 6 months. Such differences in the timing of the follow-up may partially explain these discrepancies. The prospective evaluation of the follow-up may also, in some extent, contribute to the differences noted when our study is compared with retrospective studies previously published. There are no previous studies with negative results thus far that prospectively evaluate long-term outcomes. This may be probably due to publication bias, or the fact that these biomarkers have been only recently investigated with just a few numbers of retrospective studies (only 4) published to data.

In our study, endovascular treatment (compared with microsurgery) was associated with worse outcomes, probably because the patients in our data had poorer grade at admission.

All previous studies had limitations associated with small samples, single center, retrospective nature, and systemic diseases treated with emergency management drugs that could not always be controlled ^12,17–20.^ In the present study, we used a larger sample than in the previous studies and recorded the outcomes in a prospective manner. In addition, we used more than one score for prognosis evaluation and performed a long term follow-up (6 months). Uni and multivariate statistical analysis were used to assess potential confounding factors.

### Limitations of the study

This study has advantages and flaws. A limitation of this study is that data was collected from only one center.

The interpretation of the results may have been limited by the sample size, although the sample size is larger or similar to those of other published studies. Some bias may have been inherently incorporated into observational prospective analysis and cannot be completely eliminated by the analysis of outcomes and multivariable logistic regression.

Many of these flaws may only be addressed using prospective studies with larger samples.

In conclusion, NLR or PLR at admission presented no value for the prediction of functional outcome or angiographic vasospasm risk. Further research is needed in this field—with larger prospective studies being particularly necessary.
